# Supporting breastfeeding In Local Communities (SILC): protocol for a cluster randomised controlled trial

**DOI:** 10.1186/1471-2393-14-346

**Published:** 2014-10-03

**Authors:** Helen L McLachlan, Della A Forster, Lisa H Amir, Rhonda Small, Meabh Cullinane, Lyndsey F Watson, Touran Shafiei

**Affiliations:** Judith Lumley Centre (formerly Mother & Child Health Research), La Trobe University, Melbourne, VIC 3000 Australia; School of Nursing and Midwifery, La Trobe University, Bundoora, VIC 3083 Australia; Royal Women’s Hospital, Locked Bag 300, Grattan St and Flemington Rd, Parkville, VIC 3052 Australia

**Keywords:** Breastfeeding, Infant feeding, Randomised controlled trial, Early professional breastfeeding support, Local government, Maternal and child health, Postnatal, Community-based, Drop-in centre, Home visits

## Abstract

**Background:**

Breastfeeding is associated with significant positive health outcomes for mothers and infants. However, despite recommendations from the World Health Organization, exclusive breastfeeding for six months is uncommon. Increased breastfeeding support early in the postpartum period may be effective in improving breastfeeding maintenance. This trial will evaluate two community-based interventions to increase breastfeeding duration in Local Government Areas (LGAs) in Victoria, Australia.

**Methods/Design:**

A three-arm cluster randomised controlled trial design will be used. Victorian LGAs with a lower than average rate of *any breastfeeding* at discharge from hospital and more than 450 births per year that agree to participate will be randomly allocated to one of three trial arms: 1) standard care; 2) home-based breastfeeding support; or 3) home-based breastfeeding support plus access to a community-based breastfeeding drop-in centre. The services provided in LGAs allocated to ‘standard care’ are those routinely available to postpartum women. LGAs allocated to the home-based visiting intervention will provide home-visits to women who are identified as at risk of breastfeeding cessation in the early postnatal period. These visits will be provided by Maternal and Child Health Nurses who have received training to provide the intervention (SILC-MCHNs). In areas allocated to receive the second intervention, in addition to home-based breastfeeding support, community breastfeeding drop-in centres will be made available, staffed by a SILC-MCHN. The interventions will run in LGAs for a nine to twelve month period depending on birth numbers. The primary outcome is the proportion of infants receiving *any* breast milk at four months of age. Breastfeeding outcomes will be obtained from routinely collected Maternal and Child Health centre data and from a new data item collecting infant feeding ‘in the last 24 hours’. Information will also be obtained directly from women via a postal survey. A comprehensive process evaluation will be conducted.

**Discussion:**

This study will determine if early home-based breastfeeding support by a health professional for women at risk of stopping breastfeeding, with or without access to a community-based breastfeeding drop-in centre, increases breastfeeding duration in Victorian LGAs with low breastfeeding rates.

**Trial registration:**

Australian New Zealand Clinical Trials Registry: ACTRN12611000898954.

**Electronic supplementary material:**

The online version of this article (doi:10.1186/1471-2393-14-346) contains supplementary material, which is available to authorized users.

## Background

Breastfeeding provides significant value to mothers and infants. It is the first step in the prevention of obesity, heart disease, diabetes and osteoporosis [1], and infants not exclusively breastfed are at increased risk of death from diarrhoea, pneumonia and neonatal sepsis [2, 3]. Mothers who do not breastfeed have a higher risk of ovarian and breast cancer compared to those who breastfeed [1, 4]. Breastfeeding is also beneficial to families and the community as it eliminates the need to purchase alternative infant feeding products and reduces health costs [5, 6].

The World Health Organization and Australian authorities recommend exclusive breastfeeding to six months of age [7–9] and in Australia, breastfeeding at four months is a key national indicator of children’s health, development and wellbeing [10]. However, while breastfeeding initiation in Australia is high (96% in the most recent national survey [11]), many women cease breastfeeding in the early months, or introduce infant formula in addition to breast milk. Even more striking are the increasing social disparities in breastfeeding rates over the last 20 years. Amir and Donath have demonstrated that although overall duration of breastfeeding remained reasonably constant in Australia between 1995 and 2004–05, the gap between the most and least disadvantaged families has widened considerably [12]. Infant feeding data from the 2004–2005 Australian National Health Survey showed that 66% of infants in the highest Socio-Economic Indexes for Areas (SEIFA) quintile were breastfeeding at six months, compared to 37% in the lowest [13], and data from the 2010 Australian National Infant Feeding Survey further emphasises the gaps in breastfeeding initiation and maintenance between high and low socioeconomic groups [11]. In the state of Victoria, breastfeeding rates are similar to national trends and also show marked disparities in the proportion of infants receiving *any* breast milk at six months of age in different Local Government Areas (LGAs) around the state [14]. For example, in one Victorian LGA, 68% of infants received *any* breast milk at six months of age, compared with 32% in another [15], highlighting the breastfeeding inequalities between high and low socioeconomic groups.

Given the disparities in breastfeeding across Victoria, the Department of Education and Early Childhood Development (DEECD) called for community-based interventions embedded in the LGA system that might help increase breastfeeding duration in Victorian LGAs with low breastfeeding rates [16]. A large body of evidence describes interventions which promote the initiation and/or duration of breastfeeding including several Cochrane reviews [17–21]. However very few interventions have been effective; health systems and cultural contexts are often not comparable, and interventions frequently heterogeneous [22]. Further, in the Australian context, where breastfeeding initiation is relatively high, there is little evidence to guide potential strategies to increase breastfeeding in the groups most at risk of discontinuing breastfeeding [14].

Trials conducted in Australia to increase breastfeeding include three that evaluated antenatal education programs [23–25], one which offered education and support to women both antenatally and postnatally [26], one which specifically targeted fathers [27] and four that explored postnatal strategies [28–31]. One trial increased breastfeeding initiation [25] and two increased breastfeeding at six weeks [23, 27]. No community-based trials of potentially sustainable community-level interventions were identified. Therefore in light of the lack of evidence in this area, and the interest by the DEECD to explore a community-based approach to early breastfeeding support, possible interventions including a postnatal breastfeeding support home-visiting program to provide information, encouragement and support to breastfeeding mothers and the provision of easily accessible drop-in centres where mothers could receive breastfeeding assistance have been suggested [14].

In the state of Victoria, community-based, government-funded support for new parents is provided by the Maternal and Child Health (MCH) Service, a universal primary care service for families with children from birth to school age [32]. The service is provided in partnership with the Municipal Association of Victoria (MAV), Victorian LGAs and the DEECD. The universal MCH Service offers ten consultations to parents (known as Key Ages and Stages (KAS) visits), delivered by Maternal and Child Health Nurses (MCHNs) in MCH centres located throughout all LGAs [33]. MCH centres are located in local communities, often adjacent to kindergartens, and aim to be easily accessible to parents. Victorian MCHNs are registered nurses with additional midwifery and maternal and child health qualifications. The first KAS consultation takes place at approximately one to two weeks postpartum in the mother’s home. Mothers and infants subsequently attend consultations at their local MCH centre at two, four and eight weeks; four, eight, twelve and eighteen months; and two and three and a half years of age. At each consultation, parents are given the opportunity to discuss concerns, and their child’s health, growth and development. Infant feeding outcomes are collected at KAS visits, with infant feeding practices at hospital discharge, two weeks, three months and six months postpartum reported to the DEECD.

This trial, SILC (Supporting breastfeeding In Local Communities), was designed in collaboration with the DEECD, who specifically requested an evaluation of community-based initiatives embedded in the existing Victorian MCH system to increase breastfeeding maintenance in Victorian communities. Thus SILC was designed to investigate whether a postnatal breastfeeding support home-visiting program and the provision of drop-in centres where mothers could receive breastfeeding assistance could increase breastfeeding maintenance in Victorian LGAs with low breastfeeding rates. The interventions will be delivered by MCHNs (called SILC-MCHNs) specifically employed by intervention LGAs to address breastfeeding issues women encounter. Each intervention has been pragmatically designed so that if it did increase breastfeeding rates, it could be readily incorporated into routine MCH practice in Victoria.

## Methods

### Aims

This study aims to determine whether early home-based breastfeeding support by a SILC-MCHN for women at risk of early cessation of breastfeeding, with or without access to a community-based breastfeeding drop-in centre, compared with usual/standard MCH care increases the proportion of infants receiving *any* breast milk at four months. Four months (rather than six months) was chosen as the primary outcome point because breastfeeding at four months is a key national indicator of children’s health, development and wellbeing. Given the MCH visit schedule, these data will be collected at the time of the MCH visit (the four month KAS visit).

Secondary aims of the study are to explore the proportion of infants receiving *any* breast milk at three and six months; early breastfeeding problems and women’s satisfaction with breastfeeding support; and SILC-MCHN and MCH co-ordinator views and experiences of SILC.

### Study design

This study uses a cluster randomised controlled trial (RCT) design, consisting of three arms: a comparison (standard care) arm and two intervention arms. The first intervention is early health professional home-based breastfeeding support, and the second also has the home-based support, but includes access to a community-based breastfeeding drop-in centre. The interventions will run in LGAs for a nine to twelve month period depending on birth numbers available in participating LGAs.

### Study hypotheses

#### Primary hypotheses

1. LGAs providing early home-based breastfeeding support to women at risk of early breastfeeding cessation will have a higher proportion of infants receiving *any* breast milk at **four** months compared with LGAs who provide usual MCH care.
2. LGAs providing early home-based breastfeeding support for women at risk of early breastfeeding cessation **plus** access for all women to a community-based breastfeeding drop-in centre will have a higher proportion of infants receiving *any* breast milk at **four** months compared with LGAs who provide usual MCH care.

#### Secondary hypotheses

1. LGAs providing early home-based breastfeeding support to women at risk of early breastfeeding cessation will have a higher proportion of infants receiving *any* breast milk at **three** and **six** months compared with LGAs receiving usual MCH care.
2. LGAs providing early home-based breastfeeding support for women at risk of early breastfeeding cessation **plus** access for all women to a community-based breastfeeding drop-in centre will have a higher proportion of infants receiving *any* breast milk at **three** and **six** months compared with LGAs who provide usual MCH care.

### Study population

All eligible LGAs in Victoria, Australia will be invited to participate in the trial. All eligible women who have given birth during the intervention time-frame in participating LGAs will be invited to complete a postal survey.

#### Inclusion criteria

##### Victorian LGAs

- that have a lower rate of ‘*any* breastfeeding’ at discharge from hospital than the Victorian state average [34]; and
- with more than 450 births per year.

##### For the postal survey, women

- who have given birth during the intervention time-frame in all participating LGAs.

#### Exclusion criteria

Victorian LGAs that have initiatives in place to increase breastfeeding similar to one or both of the proposed interventions.

For the postal survey, women who have given birth during the intervention time-frame in participating LGAs will not be sent a questionnaire if:

- it is known that either they or their infant have died;
- they have moved to a different LGA since giving birth; or
- they are not enrolled with the MCH service in their LGA.

### Recruitment

#### LGAs

Eligible LGAs: All eligible LGAs will be sent an Information Package including an invitation letter from the DEECD, an outline of the project and an ‘Expression of Interest’ form. Interested LGAs who sign the Expression of Interest will be briefed by the research team and asked about breastfeeding initiatives already underway.

LGAs who meet the inclusion/exclusion criteria and who agree to participate will sign a written agreement. Prior to randomisation, participating LGAs will be grouped according to size (large, medium and small) depending on numbers of births. LGAs will be allocated to the large category if they have more than 2,500 births per year, to the medium if they have between 1,000 and 2,500 births per year, and to the small category if they have fewer than 1,000 births per year. Randomisation will be stratified by these size categories. If there are more LGAs interested in participating than are required, then excess LGAs will be included in the comparison group. For example if there are four large LGAs with relatively similar numbers of births (and there are more LGAs than required) then two of these LGAs will be included in the comparison group. This process will be made explicit to LGAs in the briefings with the research team.

#### Postal survey to women

A postal survey will be sent to women who have given birth during the intervention time-frame in the participating LGAs when their infants are six months of age. To minimise the burden on the LGAs, they will be given the option of providing a contact list of women who have given birth during the intervention time-frame to a data capture/data entry service who will organise the confidential mail-out of this survey. If LGAs do not agree to provide the women’s contact details to the data capture service, the research team will prepare packages containing the questionnaire, a covering letter, and a reply paid envelope. These will be given to participating LGAs to be mailed to women directly.

Women will be informed in the covering letter that they are under no obligation to complete the survey, and that their participation will not affect any services that they receive from their MCH centre. The research team will not have access to women’s names and addresses at any stage.

### Intervention allocation

#### Randomisation procedure

**Figure 1:**
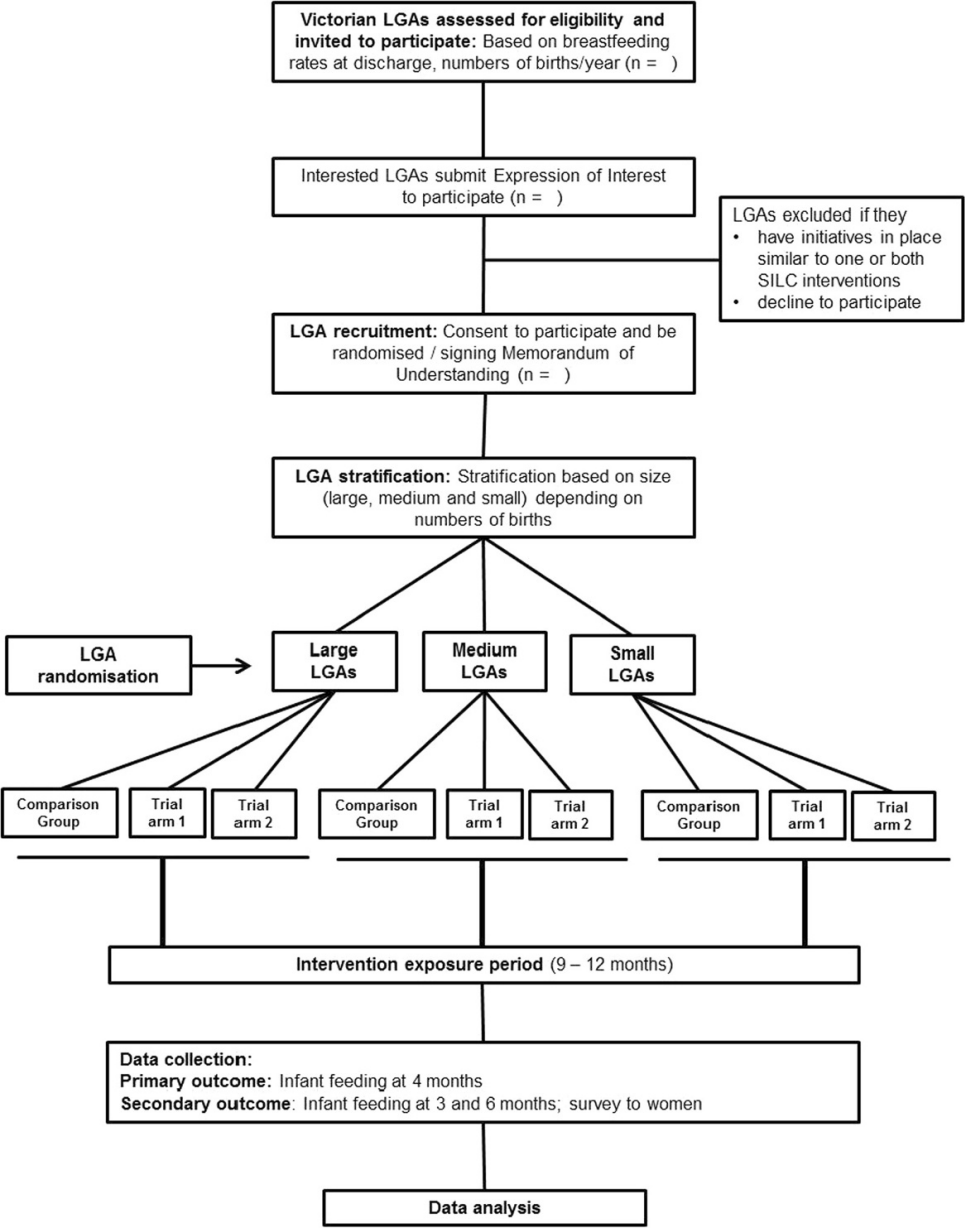
**Flow diagram to summarise SILC trial design.** LGA= Local Government Area, Comparison group= Standard MCHN care, Trial arm 1= Standard MCHN care *plus* early home-based breastfeeding support visits, Trial arm 2= Standard MCHN care *plus* early home-based breastfeeding support visits *plus* access to a breastfeeding drop-in centre. NB: Number of LGAs available for randomisation will be determined in part by number available and eligible.

Due to the nature of the intervention blinding is not possible. The unit of randomisation will be the LGA. Eligible LGAs will be randomly allocated to one of the three trial arms, stratified by the number of births (Figure 1). To ensure the process is open and transparent, allocation to trial arms will take place at a public forum to which representatives from participating LGAs will be invited. Prior to the forum the eligible LGAs will be grouped according to the number of births (large, medium, small), their name recorded on paper and placed in individual sealed opaque envelopes. An independent audience member (not linked to any of the participating SILC LGAs) will be invited to assist with the process. LGAs in the large group will be randomised first, followed by LGAs in the medium group and then the small group. The audience member will shuffle the envelopes with LGAs from the large group and select one envelope at random. This LGA will be assigned to the comparison arm. Another envelope will then be chosen by the audience member. The LGA in this envelope will be assigned to Trial arm 1 (the early home-visiting arm). The remaining envelope will then be opened. The LGA in this envelope will be assigned to Trial arm 2 (the early home-visiting plus drop-in centre arm). This process will then be repeated for the medium and for the small LGA group.

### Standard care

Services provided in LGAs allocated to ‘standard care’ are those routinely available to postpartum women in all LGAs in Victoria. A woman’s first KAS visit is conducted by a MCHN in her home, usually one to two weeks after birth. This first visit is broadly focused on early parenting and includes information about infant health and physical wellbeing, safe sleeping and sudden infant death syndrome (SIDS) as well as infant feeding [33]. Standard care includes usual support provided by MCHNs (encompassing breastfeeding assessment, support and advice as a core component of care); availability of a state-wide 24 hour MCH helpline; a 24 hour Australian Breastfeeding Association (ABA) helpline; and support by general practitioners and other health professionals as sought by families.

### Interventions

Intervention LGAs will continue to have access to all standard care, as described above.

#### Description of Trial arm 1 (early home-visiting only)

LGAs allocated to ‘Trial arm 1’ will provide early home-based visiting by a SILC-MCHN to women who are identified as being at risk of breastfeeding cessation. This is in contrast to the first KAS all women receive in their home, which is focused on parenting more generally. For women identified as being at risk of stopping breastfeeding, a SILC-MCHN home visit will be arranged during the MCH service’s first contact with the woman after hospital discharge (usually by telephone between day three and five postpartum to arrange her first KAS visit). The aim is that proactive breastfeeding assistance be provided to women as early as possible after birth. The visits will be provided by SILC-MCHNs who have received the appropriate training and education to provide the intervention (described in more detail below). The focus of the visits will be the normalisation of breastfeeding, and the provision of reassurance, with the aim of building women’s confidence to breastfeed. SILC-MCHNs will provide women with an infant feeding plan, which will also contain a list of useful websites and telephone numbers. They will also refer women to appropriate additional services as needed, and will document the assistance they provide to women.

#### Description of Trial arm 2 (early home-visiting plus access to a breastfeeding drop-in centre)

In LGAs allocated to ‘Trial arm 2’, a local community breastfeeding drop-in centre will be established in addition to SILC-MCHN early home-based breastfeeding support. The centres will be staffed by a SILC-MCHN, and where possible with a trained peer supporter or ABA community educator or counsellor.

Drop-in centres will be based in the community at places to be determined by the LGA. Ideally they will be readily accessible to women and located close to public transport where possible. They will be welcoming spaces suitable for new mothers and babies, with access to drinks, change tables, and toilets, as well as providing privacy. The aim is that women will be able to discuss breastfeeding issues or concerns with the SILC-MCHN, as well as meet other mothers to enable learning from each other. It is intended that the centres will be similar to Baby Cafés in the United Kingdom [35]. It is anticipated that the drop-in centres will open for two half days per week in small and medium LGAs and three half days per week in the large LGAs. LGAs will advertise and promote the drop-in centres within the LGA.

#### Recruitment and training of SILC-MCHNs

Funding for the SILC-MCHNs will be provided to LGAs by the DEECD, and recruitment of SILC-MCHNs will be undertaken by individual intervention LGAs. Hours of work per week will vary depending on LGA location and birth numbers.

SILC-MCHNs will be MCHNs who have previous experience in supporting mothers to breastfeed. Before the interventions commence, they will be required to attend a SILC-MCHN workshop to discuss how the interventions will be implemented. Training will be administered by the research team and an International Board Certified Lactation Consultant, and information will be provided with the aim of ensuring a consistent approach for SILC-MCHNs to offer proactive family-centred care to support women to breastfeed as long as they can. The workshop will have two objectives:

1. to provide an introduction to randomised trials and an overview of the specific trial process for SILC; and
2. to provide guidance about intervention implementation, covering topics such as: how the intervention should be provided; what is expected; protocols for identifying and supporting women with breastfeeding issues; and current best practice for common breastfeeding problems. Discussions on how to support and enable women to breastfeed will focus on providing a woman-centred supportive environment for breastfeeding [36, 37].

SILC-MCHNs and MCH co-ordinators will also be invited to attend a follow-up day two months after the start of the trial period and two additional workshops during the intervention to assess their experiences of intervention implementation. At these workshops, there will be an opportunity for input and feedback from the research team and intervention participants will be able share experiences and raise questions and concerns.

#### Determining eligibility for SILC-MCHN visit

Women will be assessed by telephone as soon as possible following discharge from hospital after birth to determine eligibility for a SILC-MCHN visit. The assessment will represent the first point of contact with the LGAs MCH services for that birth episode. It is estimated that within each intervention LGA, SILC-MCHNs will conduct an average of two home visits to approximately 30% of women who leave hospital breastfeeding. This proportion has been chosen pragmatically, both to identify those at risk of early cessation and/or experiencing problems leading to early cessation and to enhance sustainability if the intervention proves effective. There is strong evidence that infants receiving formula in the early postpartum period are at higher risk of premature cessation of breastfeeding than other infants [38, 39], and approximately 14% of Victorian-born infants are being supplemented with infant formula in the early days postpartum [34]. It is also known that some 30% of women self-identify breastfeeding problems in the first few days postpartum [40], though on its own this is unlikely to be a reliable indicator for risk of early cessation. Therefore we aim to identify and conduct visits to the 30% of women who are most at risk of early breastfeeding cessation, using a mix of eligibility criteria.

An assessment tool will be developed and tested in non-trial LGAs to determine eligibility for a SILC-MCHN visit. The aim is to provide proactive support to the women most likely to cease breastfeeding. A visit will be arranged:

- if a woman’s infant has received any formula as well as breast milk, either expressed breast milk (EBM) and/or at the breast, in the 24 hours prior to telephone contact; or
- if a woman is distressed about breastfeeding or asks for help with breastfeeding when telephoned, even if she is not supplementing with formula or giving EBM. MCHNs may also identify women for a SILC-MCHN home visit at a later standard MCH visit, if needed.

If formula use alone identifies less than 25% of women who leave hospital breastfeeding, EBM use in the 24 hours prior to telephone contact (whether or not infant formula has been given) will be included in the assessment criteria, as this is also known to be a risk factor for early breastfeeding cessation [41].

### Process evaluation

A number of strategies are planned to monitor intervention uptake, assess adherence to protocols and describe experiences with trial implementation.

#### Measures of intervention exposure

SILC-MCHN home visits: number of women identified as eligible; number of women who decline a visit; number of visits; timing of visits; and time spent during visits.

Drop-in centres: number of women who visit drop-in centres, reason for the visit and total number of visits to each drop-in centre.

#### Adherence to protocols

SILC-MCHNs will keep a log of what is provided to women during visits. Breastfeeding concerns women raise or issues identified by the providers will be documented on pre-coded data sheets at home visits and drop-in centres.

SILC-MCHNs will attend a total of four workshops during the trial period: one before implementation of the interventions and three while the interventions are in place to discuss adherence to SILC protocols, review processes and discuss any concerns SILC-MCHNs may have. Research team members will visit LGAs at regular intervals (estimated to be 3–5 times) during the intervention period, to monitor protocol adherence and discuss concerns about intervention implementation with SILC-MCHNs and MCH co-ordinators.

#### Intervention implementation evaluation by SILC-MCHNs and MCH co-ordinators

An evaluation of how the interventions have been carried out will be undertaken using surveys and focus groups with SILC-MCHNs and MCH co-ordinators. SILC-MCHNs attending the final SILC workshop at the conclusion of the intervention will be invited to participate in focus groups and complete a structured questionnaire including open-ended questions. The questionnaire will explore their views and experiences of being a SILC-MCHN. They will be able to report on any highlights, any problems or barriers in implementation of the interventions, and to make recommendations on the improvement of intervention implementation. There will be two focus groups led by the research team – one group for SILC-MCHNs from Trial arm 1 and the other group for SILC-MCHNs from Trial arm 2. Focus groups will give SILC-MCHNs an opportunity to reflect on and discuss their experiences of participating in the SILC interventions.

Face-to-face interviews will be conducted with MCH co-ordinators and managers from intervention LGAs. These interviews will provide an opportunity for participants to share their experiences and to provide information regarding problems or barriers to the introduction and embedding of the interventions, which will be important in terms of sustainability if the interventions are shown to be effective.

### Sample size

Infant feeding outcomes are routinely collected by the MCHNs at KAS visits, and include infant feeding at hospital discharge, two weeks, three months (asked at the four months KAS visit) and six months postpartum (asked at eight months KAS visit). Given the non-contemporaneous nature of ascertainment of the infant feeding outcomes and that breastfeeding at four months is a key national indicator of children’s health, the four month KAS visit was determined the most appropriate time to collect infant feeding outcome data. The standard MCH questions related to infant feeding have the potential for misclassification of infant feeding outcomes, therefore a new question on infant feeding will be added to the data collected at the four month KAS visit that provides a cross-sectional snapshot of the percentage of infants receiving *any* breast milk (‘in the last 24 hours’). Our primary outcome measure is thus the percentage of infants receiving *any* breast milk at four months.

There are no local representative data on infant feeding outcomes at four months, so we estimated the four month figures from three and six-month state-wide infant feeding data. We plan to include only LGAs with breastfeeding rates below the state average at hospital discharge and with more than 450 births per annum, so we used the most recent data available (2008/2009) to identify potentially eligible LGAs and calculate their breastfeeding rates [34]. The state average for *any* breastfeeding at hospital discharge was 87%, and for the LGAs whose rate was less than the state average, the average rate of *any* breastfeeding at hospital discharge was 81%. We then used the rate of *any* breastfeeding at three months in this group of LGAs (53%) and any breastfeeding at six months (39%) to estimate a figure for four months. We consider the drop is likely to be continuous, so estimate that 48% are giving *any* breast milk at four months.

While the unit of randomisation is the LGA, the MCH centres form the clusters. We aim to have the power to detect an increase in the overall breastfeeding rates at four months from 48% to 58%. With this change and assuming a standard error for the average breastfeeding rate in each LGA of 5%, sample size estimates show that we need approximately 4 LGAs in each trial arm. To achieve this standard error we need to have 374 women in each arm (without allowing for clustering). Given that we will be obtaining breastfeeding data on all women in each LGA, with an estimated average of about 1000 births per LGA per annum, we can obtain the required sample size allowing for an inflation factor of approximately 2.7 (1000/374), which if the *geometric* mean number of MCH centres is 86 (calculated on data from MCH centres in the potentially eligible LGAs), allows for an intra-cluster correlation (ICC, ρ) of 0.02 (2.70= 1 + 85*ρ). We could not identify relevant literature on which to base our estimate for the ICC in breastfeeding trials, therefore we used the conservative estimate of 0.02 which is larger than ICCs found in cluster trials for smoking cessation in pregnancy [42]. This was also consistent with the variation we found in state-wide MCH data.

Calculating the number of women who will be eligible to potentially receive the intervention is based on the fact that the proposed intervention will only have the ability to affect women who are breastfeeding at discharge, so to gain this 10% increase overall (i.e. 48% to 58%) we will need to increase the rate of breastfeeding from 59% (or 48%/81%*100) to 72% (58%/81%*100) among those who are breastfeeding at hospital discharge. For a simple random sample this would require 224 women per each intervention trial arm (with alpha 0.05 and 80% power), i.e. 448 in total. Taking into consideration the potential effect of clustering we have inflated the sample size assuming an intra-cluster correlation (rho) of 0.02 (as discussed above), so we require approximately 400–500 women in each intervention arm.

### Data collection

The data items for each component of the study are summarised in Table 1. The interventions will run in LGAs for a nine to twelve month period depending on birth numbers available in participating LGAs. The first two months of the interventions will be a pilot phase/run-in period. This will give SILC-MCHNs the opportunity to provide feedback on issues they may encounter and the research team the opportunity to refine protocols. Data collected during the run-in period will not be used in the final analysis.

**Table 1:** **Data collection for SILC**

Data collection time-point	SILC-MCHN visit assessment	SILC-MCHN visit	Drop-in centre visit	4 month KAS visit	8 month KAS visit	SILC postal survey	IV close
**Maternal data**							
Date of birth	X	X					
Age				X		X	
Parity		X	X	X		X	
Health care card status				X		X	
Income level						X	
Aboriginal/Torres strait islander status				X		X	
Marital status						X	
Highest education attainment						X	
Smoking status (current and in pregnancy)						X	
Alcohol consumption (current)						X	
Maternal height and weight						X	
Employment status						X	
Country of birth				X		X	
Year arrived from overseas				X		X	
Residency status						X	
Current postcode of residence			X			X	
Breastfeeding intention						X	
Infant feeding support after hospital discharge						X	
Breastfeeding complications after hospital discharge						X	
Reasons for ceasing/not commencing breastfeeding						X	
Knowledge and use of community infant feeding services						X	
**Paternal data**							
Country of birth				X			
**Birth data**							
Method of birth				X		X	
**Infant data**							
Date of birth	X	X		X		X	
Gestational age at birth				X			
Infant age			X	X	X	X	
Infant feeding ‘in the last 24 hours’	X	X		X		X	
Infant feeding at three months of age				X			
Infant feeding at six months of age					X		
Infant age at breastfeeding cessation						X	
Infant age at introduction of infant formula						X	
Infant age at introduction of solids						X	
**MCH centre data**							
MCH centre postcode				X	X		
**Process evaluation data**							
Number of women assessed, eligible for, received and declined SILC-MCHN visit	X						
Length of SILC-MCHN visits		X					
Topics discussed, factsheets provided and website information provided during SILC-MCHN visits		X					
Reason(s) for visiting SILC drop-in centre			X				
First or subsequent visit to SILC drop-in centre			X				
**SILC-MCHN data**							
Evaluation of SILC trial via focus groups and surveys							X
**MCH co-ordinator data**							
Evaluation of SILC trial via interviews							X

KAS= Key Ages and Stages visit, IV= intervention, MCH= Maternal and Child Health, SILC-MCHN= SILC Maternal and Child Health Nurse.

#### Baseline data

Baseline breastfeeding rates will be collected to allow a before and after analysis of breastfeeding rates in each LGA, and to assess comparability of trial arms at trial commencement. Baseline breastfeeding outcomes will be collected for a period of three months before infants exposed to the interventions will have their routine MCH appointments (when breastfeeding outcomes are collected). These data will be obtained from routinely collected MCH centre data (three, four and six month infant feeding data) and from a new data item to collect infant feeding ‘in the last 24 hours’ (in all participating LGAs). Inclusion of this new data item will give higher quality cross-sectional data, enhancing the capacity to discern changes that may arise from the intervention. It will be completed by MCHNs with mothers at their routine four month KAS visit.

#### Outcome data

There will be two components to outcome data collection. Breastfeeding outcomes will be obtained from routinely collected MCH centre data and from the new data item collecting infant feeding ‘in the last 24 hours’ at four months.

In addition, information will also be obtained directly from women via a postal survey. All women who give birth during the intervention time-frame in the participating LGAs will be sent a questionnaire when their infants are six months of age, with the exception of those who meet the exclusion criteria described previously. Data will include socio-demographic details and infant feeding outcomes (*any* breastfeeding at survey completion, and duration of *any* and *only* breastfeeding). Women will be asked if they have had difficulties with breastfeeding, and if and where they obtained support or help with breastfeeding. Information will also be collected about home visiting (as part of the intervention or other home visiting services) and breastfeeding services (including the intervention drop-in centres). Women will be given the opportunity to report on their views and experiences of the available services.

#### Outcome variables

The primary outcome of the study is *any* breastfeeding at four months. A single data item asking about *infant feeding* ‘in the last 24 hours’ will be used to ascertain this outcome at the routine four month KAS visit. The four month KAS participation rate from the last state-wide report was 93.6% [43]. Three and six month infant feeding outcomes will be obtained from routinely collected MCH centre infant feeding data collected at the four month and eight month KAS visits respectively. The eight month KAS participation rate from the last state-wide report was 85.6% [43].

Other variables of interest (or potential confounders for the primary outcome) which will be abstracted from routinely collected MCH data are:

- Gestational age of infant at birth;
- Date of birth of infant;
- Parity of mother;
- Mode of birth (vaginal, caesarean delivery);
- Age of mother;
- Date of the routine four month KAS visit
- Infant age in weeks at the routine four month KAS visit;
- Health Care Card [proxy for socioeconomic status];
- Aboriginal/Torres Strait Islander status of mother;
- Country of birth of mother;
- Year mother arrived from overseas (where relevant);
- Country of birth of father; and
- MCH centre postcode.

For the secondary aims of the study, addressed via the survey exploring women’s satisfaction with the breastfeeding support they received, the following variables will be collected:

- Infant feeding at six months;
- Introduction of solids;
- Infant feeding intention;
- Breastfeeding complications, such as milk supply issues, nipple pain and mastitis;
- Reasons for not commencing breastfeeding;
- Reasons for ceasing breastfeeding;
- Breastfeeding support after discharge (domiciliary visits, Lactation Consultant, MCHN, SILC-MCHN, MCH line, ABA, breastfeeding drop-in centre);
- Woman’s knowledge of community breastfeeding services available (SILC-MCHN, breastfeeding drop-in centre); and
- Background data such as maternal age, marital status, education, income level, employment status, smoking status, and maternal height and weight.

Intervention implementation – process and impact measures:

- Assessment of adherence to intervention protocols;
- Evaluation of the SILC trial via surveys and focus groups with SILC-MCHNs; and
- Interviews with MCH co-ordinators.

### Data analysis

Data from SILC-MCHN and drop-in centre logs will be entered into a Microsoft Access database [44]. Data from questionnaires will be entered by a data-entry company. All data will be analysed using Stata Version 13 [45].

#### Primary outcome

In relation to the trial hypotheses, each intervention group will be compared to the standard care group using intention to treat analysis. For the primary outcome, proportions of women giving their baby *any* breast milk at four months will be compared using odds ratios derived using logistic regression. A multivariate analysis will be carried out to adjust for known potential confounders, as well as for the baseline breastfeeding rates of each LGA. In all analyses, adjustment will be made for clustering at the MCH centre level, and the LGA strata (i.e. LGA size) will be taken into account. Results will be presented as adjusted odds ratios, with 95% confidence intervals.

#### Secondary outcomes

All comparisons that involve percentages of breastfeeding will be undertaken using the same method as the primary outcome, with each intervention group compared to the standard care group using intention to treat analysis. Comparison of means will be undertaken for continuous variables such as breastfeeding duration using t-tests where data were normally distributed or medians compared otherwise using Mann-Whitney U tests.

Data obtained from women’s surveys will be explored using descriptive statistics. Analysis for pre-coded responses will be undertaken using descriptive statistics, and frequencies and percentages presented. Outcomes will be presented by trial arm; with statistical comparisons undertaken when appropriate using the chi-square test. Women’s responses to open-ended questions will be analysed inductively and grouped into analytical descriptive categories [46]. The categories will be discussed by the research team and final themes agreed. Comparisons will be made between trial arms where possible.

Data from the focus groups with SILC-MCHNs and face-to-face interviews with MCH co-ordinators or managers will be transcribed and grouped into analytical descriptive categories using basic thematic analysis [46]. SILC-MCHN survey data will be analysed using a mix of quantitative (for pre-coded responses) and qualitative (for open-ended responses) methods.

### Timelines

**Figure 2:**
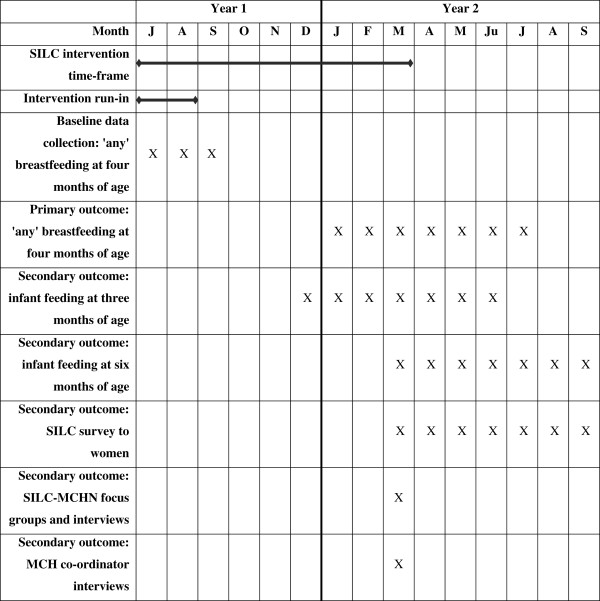
**Timeline estimates for the SILC trial.** The figure gives timeline estimates for the intervention running for a nine month period in SILC LGAs. The first two months will be a run-in period. Data collected during the run-in period will not be used in the final analysis.

The project, from design of the interventions to data collection and analysis is expected to take three years. Figure 2 shows the timeline estimates.

### Ethical considerations

#### Consent

LGAs will sign a written agreement to participate and be randomised. For the postal survey, women will be provided with information about the study in a covering letter mailed to them with the survey, and consent will be implied by the completion and return of the questionnaire.

#### Ethics approvals

Approval for the study has been granted by:

- La Trobe University Human Ethics Committee (project number 11–068);
- The Department of Education and Early Childhood Development (project number 2011_001305).

#### Risks/inconveniences/benefits

It is not anticipated that this trial poses any risks to LGAs or to women who participate. It is possible that women will benefit from the interventions, but this is unknown. While some women with breastfeeding difficulties may experience stress associated with their breastfeeding problems, MCHNs in both intervention and comparison areas have both the skill set necessary and the knowledge of available support services in order to address these issues.

The postal survey sent to women will contain contact details of support services available to them. The contact details of the research team and the La Trobe Ethics Committee will also be provided. Every effort will be made to ensure that women who should not receive a survey (as per the exclusion criteria) are not sent one.

### Dissemination

At trial completion, a final report will be submitted to the DEECD. All LGAs will be de-identified in this report, a summary of which will be available on their website. All participating LGAs will receive a separate report of outcomes for women in their municipality, including postal survey responses. SILC presentations will also be undertaken in each LGA following trial completion, and at both national and international conferences.

### Study administration

#### Research team

##### Chief Investigators

Associate Professor Helen McLachlan

Professor Della Forster

Associate Professor Lisa Amir

Professor Rhonda Small

#### Statistician

Dr Lyndsey Watson

##### Project Co-ordinators

Dr Meabh Cullinane

Dr Heather McKay (maternity leave replacement)

#### Research Officer

Dr Touran Shafiei

### Project governance

#### Meetings

It is anticipated that the research team will meet fortnightly throughout the project, and more often as necessary. The research team will also meet regularly with the DEECD.

#### Study advisory group

An advisory group will be established to bring together a group of people with relevant expertise and interest in the trial. The group will contribute ideas and advice to the research team through all stages of the project, comment on drafts of materials and resources developed to support the project (e.g. questionnaires, reports), participate in discussion of the findings and their implications for future research and implementation strategies and assist in developing appropriate strategies for disseminating the findings of the project. Terms of Reference have been developed (see Additional file 1). Overall responsibility for the conduct of the research, analysis of the data and publication of the findings remains with the research team.

### Trial status

Twenty one Victorian LGAs were eligible to participate in SILC. Eighteen of these LGAs submitted a written Expression of Interest to participate. Following briefing sessions with these 18 LGAs, four were deemed ineligible to participate as they had ongoing initiatives in place similar to one or both of the interventions. A further four declined to participate. The remaining ten LGAs, comprising three large, three medium and four small LGAs, agreed to participate and be randomised. During randomisation, one LGA from each group (small, medium, large) was randomly assigned to be the comparison LGA for that group, one was assigned to receive home-visits by a SILC-MCHN, and one assigned to received home-visits and drop-in centre access. Because there were four LGAs in the small group, two LGAs were randomly assigned to be comparison LGAs.

The interventions commenced in LGAs in July 2012 and ran for a nine month period until March 2013. Data collection is now complete. Analysis and final writing up is underway. Final results are expected in 2015.

## Discussion

Breastfeeding initiation is high in Australia, but many women stop in the early months postpartum, especially those in lower socioeconomic communities. We have designed this trial to address this by situating the study in areas with low breastfeeding rates and testing two strategies with the potential to provide support for women in the community to maintain breastfeeding. If shown to be effective, the intervention programs have been pragmatically designed so that if either increased the maintenance of breastfeeding, then incorporation into practice across the state should be readily achievable.

## Electronic supplementary material

Additional file 1:**SILC Advisory Group Terms of Reference.**(DOCX 18 KB)

## Abbreviations

ABA: Australian Breastfeeding association
DEECD: Department of Education and Early Childhood Development
EBM: Expressed breast milk
KAS: Key Ages and Stages
LGA: Local Government Area
MAV: Municipal Association of Victoria
MCH: Maternal and Child Health
MCHN: Maternal and Child Health Nurse
SILC: Supporting breastfeeding In Local Communities
SILC-MCHN: SILC Maternal and Child Health Nurse.
