# Supplementary material for: Supporting breastfeeding In Local Communities (SILC): protocol for a cluster randomised controlled trial
Source: BMC Pregnancy Childbirth. 2014 Oct 3;14:346. doi: 10.1186/1471-2393-14-346 (PMC4287548; doi:10.1186/1471-2393-14-346)
Supplement: Supplementary file 1 — Additional file 1:SILC Advisory Group Terms of Reference.(DOCX 18 KB) [file 12884_2013_1270_MOESM1_ESM.docx]

**Advisory Panel**

**Terms of Reference**

The Victorian Government supports and promotes breastfeeding as an important determinant of maternal and child health and wellbeing. Their commitment to improving breastfeeding rates includes the development of the Victorian Breastfeeding Action Plan. The Victorian Breastfeeding Research Project Phase Two [Supporting breastfeeding in Local Communities] is part of this action plan. Supporting breastfeeding in Local Communities (SILC) will assess strategies to increase the maintenance of breastfeeding in Victoria.

The Child and Adolescent Health and Wellbeing Division within the Department of Education and Early Childhood Development (DEECD) has commissioned A/Prof Helen McLachlan, Prof Della Forster, Dr Lisa Amir and Prof Rhonda Small from Mother & Child Health Research, La Trobe University to undertake this project. SILC will take three years and conclude in July 2014.

The research will consist of a three arm cluster randomised trial. It aims to explore whether early home-based breastfeeding support by a Maternal and Child Health Nurse (MCHN) for women with identified breastfeeding issues, with or without access to a community-based breastfeeding drop-in centre, increases the proportion of infants who receive any breast milk at four months. The project will be conducted in local government areas (LGAs) with low breastfeeding rates.

The SILC Advisory Group is established to bring together a group of people from a range of backgrounds, with relevant expertise and/or an interest in the trial to:

1. Contribute ideas and advice to the research team through all stages of the project;

2. Comment on drafts of materials and resources developed to support the project (e.g. questionnaires, reports);

3. Participate in discussion of the findings and their implications for future research and implementation strategies;

4. Assist in developing appropriate strategies for disseminating the findings of the project.

Responsibility for the conduct of the research, analysis of the data and publication of the findings remains with the research team.

**Meeting frequency and dates**

The Advisory Group will meet 3 monthly.

**Operating principles**

Confidentiality: Members are encouraged to be open and candid in discussing items at Advisory Group meetings. For this reason, members will flag information which is confidential and all members will agree to maintain confidentiality around these issues.

**Communications with members will include:**

• Meetings as scheduled;

• Reading materials and draft agendas for forthcoming meetings to be emailed to members;

• Email reminders of upcoming meetings;

• Progress reports from the research team intermittently throughout the trial.
